# Rhino-orbital mucormycosis: Our experiences with clinical features and management in a tertiary care center

**DOI:** 10.22336/rjo.2021.69

**Published:** 2021

**Authors:** Satya Prakash Singh, Jagriti Rana, Vinod Kumar Singh, Rupanshi Singh, Reena Sachan, Shivangi Singh, Sachin Jain

**Affiliations:** * Department of Ophthalmology, Moti Lal Nehru Medical College, Prayagraj, Uttar Pradesh, India; ** Department of Microbiology, Moti Lal Nehru Medical College, Prayagraj, Uttar Pradesh, India; *** Department of Otorhinolaryngology, Moti Lal Nehru Medical College, Prayagraj, Uttar Pradesh, India

**Keywords:** Rhino-orbital-cerebral, mucormycosis, amphotericin, diabetes, Covid-19

## Abstract

**Objective:** To determine the prevalence, risk factors, and elaborate our experiences with diagnosis and treatment of patients with mucormycosis, enabling a better understanding of the disease and its management.

**Methods:** This is a case series of patients with Covid-19 associated with Rhino-orbital-cerebral mucormycosis, managed in our tertiary care center from April 2021 to June 2021.

**Results:** Six cases of Covid-19 associated with Rhino-orbital-cerebral mucormycosis have been analyzed in the study. The mean age of patients was 40.67 years with a male preponderance (83.3%). The most common complaint was headache (100%), while a minority (33%) came with ocular complaints. All the patients either had a previous history of diabetes mellitus or developed increased blood sugar levels following Covid infection, and were kept on insulin to control their blood sugar levels. 4 patients (66.67%) had a history of corticosteroid use during Covid-19 hospitalization. Treatment included intravenous liposomal Amphotericin B (100%), functional endoscopic sinus surgery (66.67%), maxillectomy (33.33%) and transcutaneous retrobulbar liposomal Amphotericin B (33.33%). Amphotericin B induced nephrotoxicity, which was seen in 1 patient (16.67%). Mortality occurred in only one patient (16.67%), 25 days following successful surgery.

**Conclusion:** Diabetes Mellitus is the most important predisposing factor for the development of Covid-19 associated Rhino-orbital-cerebral mucormycosis. Early presentation, prompt diagnosis and timely initiation of treatment with liposomal Amphotericin B and surgical debridement along with strict blood sugar control can lead to a favorable outcome. However, regular follow-up and monitoring of serum electrolytes and kidney profile must be ensured for such patients.

## Introduction

Mucormycosis is an extremely fatalistic and a very fulminant form of Zygomycosis caused by Mucorales species of the phylum Zygomycota, causing serious opportunistic infections. It was first described by Paultauf in 1885 [**1**]. It is the third most common angio-invasive fungal infection, after candidiasis and aspergillosis [**2**]. The fungus gains access to the deeper tissues rapidly in the immunocompromised and especially in the diabetics (60-80%) [**3**], although haematological diseases, neoplasias, chronic renal failure, anti-neoplastic agents, burns, malignancies like lymphomas and leukemias, long-term corticosteroid and immunosuppressive therapy, protein-energy malnutrition, burns and acquired immune deficiency syndrome (AIDS) are some important predisposing factors [**4**]. 

In the second wave of Covid-19, India has witnessed a dramatic increase in mucormycosis infection in post-Covid-19 patients. Currently, in the COVID-19 pandemic, secondary infections are reportedly very common (10-30%) in hospitalized, severely ill COVID-19 patients, with fungal infections being 10 times more common than others. As a result of indiscriminate steroid usage in a patient with COVID-19 to check the progression of respiratory failure [**5**], there can even be a manifestation of latent DM, increased secondary infections and modulation of the host’s immune system, rendering the host susceptible to develop opportunistic infections like mucormycosis. It is still under exploration whether the virus itself interferes with insulin secretion and thus, the glycemic control. Besides the already acknowledged role of diabetic ketoacidosis (DKA), iron metabolism and hyperglycemia, some new factors are being taken into consideration, which may be attributed to a surge in mucormycosis in post-Covid patients, such as the role of ferritin, high serum iron, free radical induced endotheliitis, hepcidin activation and upregulation of glucose receptor protein (GRP78) [**6**].

The incidence of mucormycosis globally varies from 0.005 to 1.7 per million of population [**7**]. In India, its prevalence is 0.14 per 1000, approximately 80 times higher than in the developed countries [**8**]. In India, rhino-orbital-cerebral presentation with uncontrolled DM is the predominant characteristic [**9**].

Mucor sinusitis can progress very rapidly from involving the sinuses to extending into the orbits leading to varied presentations like headache, pain, ophthalmoplegia, ptosis, proptosis, and eventual blindness [**10**]. Around 80% of cases rapidly develop an intracranial extension and the progressive angio-invasion can lead to cavernous sinus thrombosis and cerebrovascular accidents [**10**]. The fatality rate prevalent world-wide is 46% [**11**]. However, an intracranial involvement or an orbital involvement can increase the fatality rate to as high as 50% to 80% [**12**].

Early diagnosis and early commencement of multidisciplinary treatments by an Otolaryngologist, an Ophthalmologist, and an Infectious Disease Specialist after reviewing the patient and aggressively managing by surgical and medical treatment can be life saving for these patients. 

We present a series of patients, all of whom had diabetes, who were hospitalized in our center and were managed surgically and with liposomal Amphotericin B treatment, administered intravenously.

## CASE 1

A 51-year-old housewife and a known diabetic for the past 10 years, presented to the post- Covid facility of our hospital on 17th May, 2021. Five days prior to her presentation to our facility, she noticed a left sided facial swelling, loss of sensations over the left cheek, black crusting, and blackish discharge at the outer end of the left nostril along with nasal stuffiness and a few ulcers on the upper part of the mouth. Three days before presentation, she also noticed a drooping of her left upper eyelid and developed persistent headache. 

She was diagnosed as Covid-19 positive on Real-time Polymerase Chain Reaction (RT-PCR) done on 24th April, 2021, after which she underwent a 2-week in-patient treatment in the Covid ward of our hospital. A repeated RT-PCR done on 8th May, 2021, was negative and she was discharged, only to be readmitted on 17th May, in the post-Covid ward with the chief complaints enlisted above. There was no history suggestive of Covid vaccination or intake of steroids.

The investigations at the time of her admission were: Random Blood Sugar (RBS) was 506 mg/ dl (below 200 mg/ dl), Serum Na+ was 134.2 mmol/ L (136-145 mmol/ L), Serum K+ was 4.22 mmol/ L (3.6-5.2 mmol/ L), and Serum Ca2+ was 0.83 mmol/ L (1.15-1.30 mmol/ L), suggesting borderline hyponatremia and hypokalemia. Her renal function tests (RFT) and her coagulation profile were within normal limits. Neutrophil count was 85% (40-80%) and moderate lymphopenia 9% (20-40%) was present. High Resolution Computed Tomography (HRCT) of the thorax showed the CT lung score of 17/ 25.

**Fig. 1:**
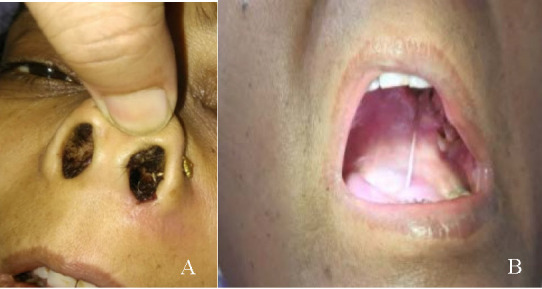
**A.** Blackish debris in the anterior part of the left nostril. **B.** Necrotic lesion on the hard palate

Physical examination showed blackish crusts and debris in the anterior part of the left nostril (**Fig. 1A**). Deep nasal swab was sent for KOH staining and for culture and sensitivity. Oral cavity examination showed ulcerated lesions, a necrotic patch on the hard palate of around 5 cm × 5 cm in size (**Fig. 1B**). Tissue biopsy from this area was sent for histopathology.

**Fig. 2:**
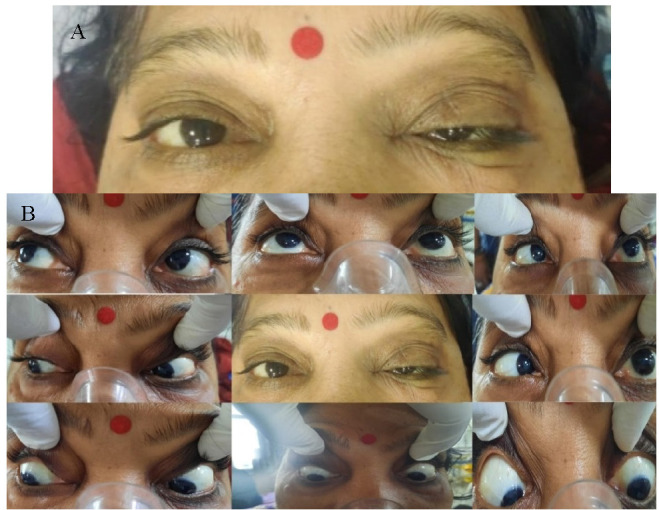
**A.** Severe ptosis of the left eyelid. **B.** Restriction of left abduction movement

Diagnostic nasal endoscopy revealed necrosis of the left inferior turbinate and blackish debris present in the nasal cavity and marked pallor of the palate of left side. There was severe ptosis of the left eye (LE) (**Fig. 2A**) and restriction of the left abduction movement (**Fig. 2B**). The visual acuity was 20/ 40 in the right eye (RE) and 20/ 60 in the LE with brisk pupillary reaction in both eyes. A superficial haemorrhage was present, 1/ 3rd disc diameter away along the superotemporal arcade. The corneal sensation in the LE was markedly diminished and hypoesthesia was present on the left side of the face.

**Fig. 3:**
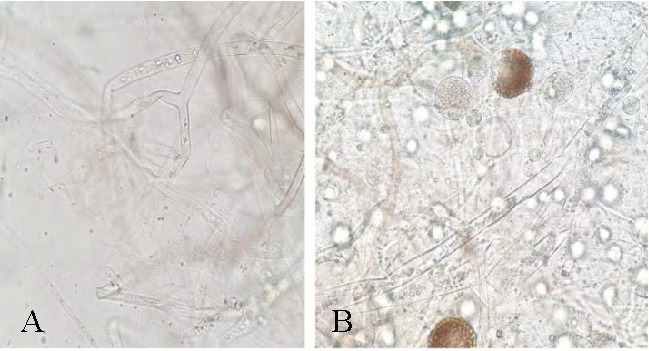
**A** and **B** KOH mount images

**Fig. 4:**
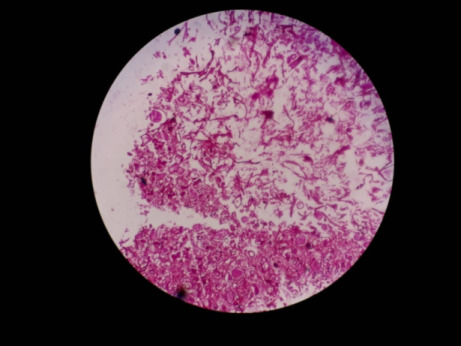
PAS section - pink colored hyphae branching at right angles, and interspersed with inflammatory cells

The KOH mount showed aseptate, broad hyphae with branching at right angles suggestive of mucorales (**Fig. 3 A, B**). Histopathology revealed stained section (**Fig. 4**) pink colored aseptate fungal hyphae and areas of necrosis interspersed with inflammatory cells and epithelioid cells in the Periodic acid-Schiff (PAS).

**Fig. 5:**
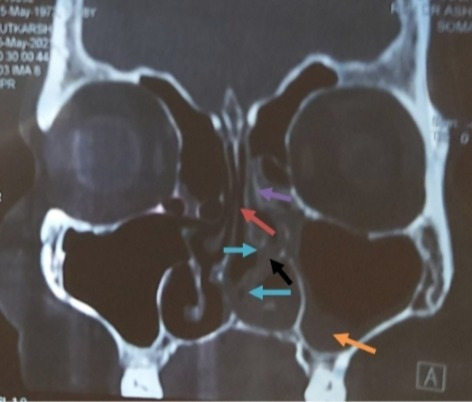
Coronal section of CT PNS showing occlusion of left maxillary ostium (black arrow), moderate mucosal thickening in the left maxillary sinus with air-fluid level (orange arrow), moderate mucosal thickening of the left ethmoidal sinus (purple arrow), nasal septum deviated to right side (red arrow), mucosal hypertrophy of bilateral inferior and middle turbinates (left more than right shown by blue arrows)

**Fig. 6:**
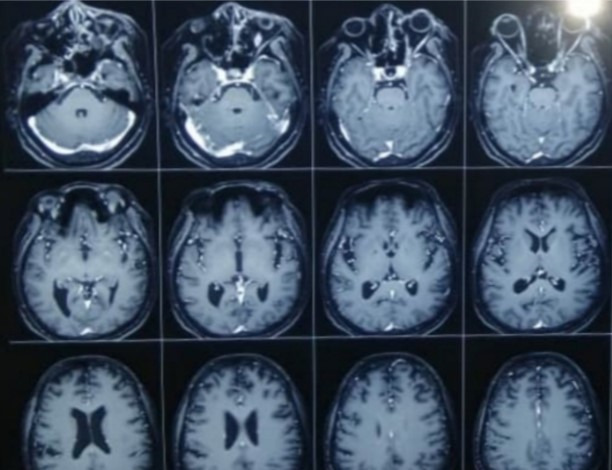
Contrast enhanced MRI of brain, orbits and PNS showing no intracranial or intraorbital involvement

CT Scan of Paranasal sinuses (PNS) revealed osteolytic and osteopenic changes in the left anterior part of the hard palate, moderate left maxillary sinusitis, and mucosal thickening of left maxillary sinus ostium (**Fig. 5**). Contrast-enhanced Magnetic Resonance Imaging (CEMRI) of brain and PNS revealed no intraorbital or intracranial extension of the lesion (**Fig. 6**). It also showed involvement of the left half of nasal cavity, medial wall and the floor of the maxillary sinus and extension of lesion into the left infratemporal fossa.

**Fig. 7:**
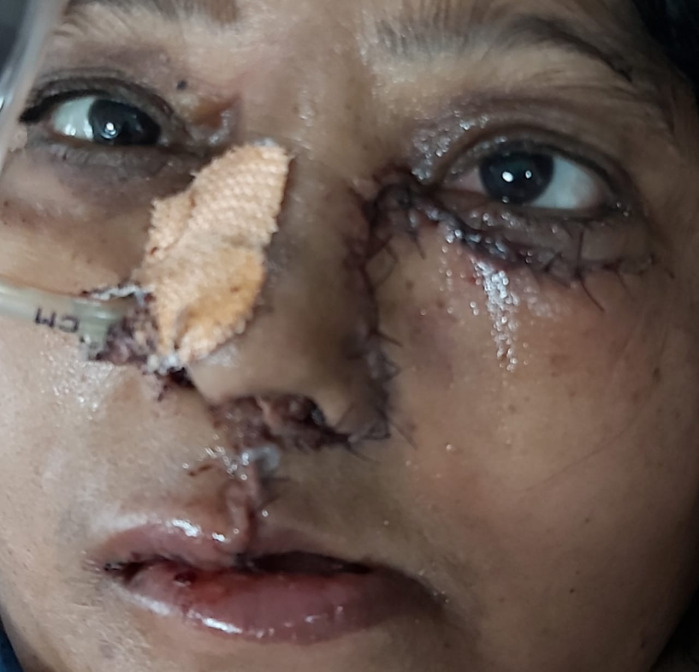
Second post-operative day

Based on these findings, a provisional diagnosis of mucormycosis was made. She was started on insulin infusion, intravenous (i.v.) antibiotics, and injection Liposomal Amphotericin B (AMB) was started intravenously. Left extended palatal maxillectomy was done. Post-surgery, injection Liposomal AMB was continued. The patient showed good recovery (**Fig. 7**) with stable vitals and a controlled DM. A complete resolution of the ptosis was observed in the left eye. Her serum electrolytes and RFT were monitored daily. She was discharged after 15 days of surgery, as per her request. She came for 2 follow-up visits in the OPD. An artificial denture was placed and she could have semi-solid diets. Sadly, she died 25 days following surgery, in a private hospital of the city, due to cardiopulmonary arrest.

## CASE 2

The patient, a 42-year-old male, and a known diabetic for 3 years, well controlled on oral hypoglycaemic drugs (Glicazide 60 mg sustained release), presented to our post-Covid facility on 5th May, 2021, with facial swelling, toothache, loss of 1 tooth, and headache for 4 days. He had been Covid Positive on RT-PCR done on 16.04.2021. He was admitted in our Covid ward for 10 days, during which he was kept on supplemental oxygen therapy and injection Dexamethasone intravenously, as per his admission records. His RT-PCR came negative on 26th April, 2021, and he was discharged. On discharge, he was advised to take Tablet Medrol (8 mg Methylprednisolone) for one week.

Upon admission, his RBS parameters were 240 mg/ dl, lymphocytes were 18.9%, HIV 1, HIV 2 and Anti-HCV Antibody were non-reactive, RFT and serum electrolytes were normal. CRP was reactive, Serum LDH was 447.6 U/ l (240-480 U/ l), LFT was normal, D-dimer was 0.5921 mcg/ ml (<0.5 mcg/ ml), coagulation profile was normal, HbA1c was 7.1 (<6) and lipid profile was normal. He had no history of Covid vaccination.

**Fig. 8:**
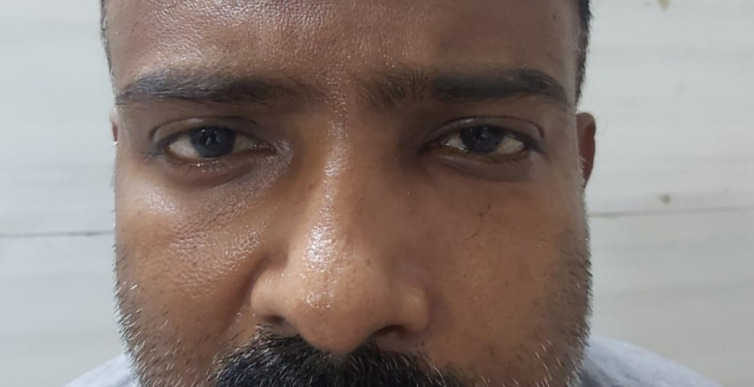
Left sided facial puffiness

**Fig. 9:**
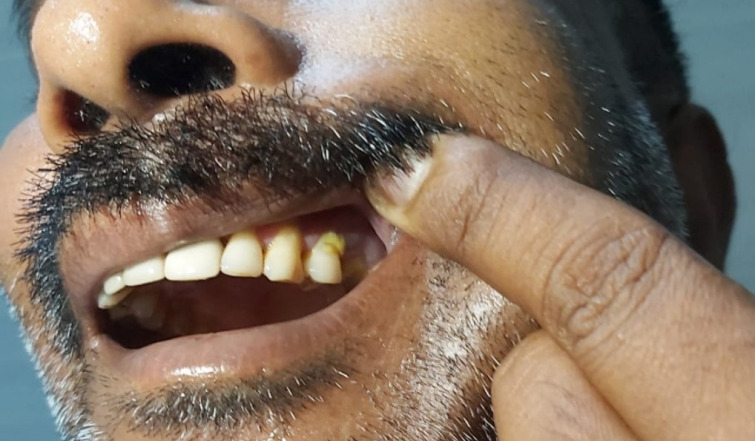
Absence of upper left premolar

Upon physical examination, there was mild, diffuse, tender swelling, over the left half of the face (**Fig. 8**). Nasal endoscopy showed necrosis of the left inferior turbinate, and crusty discharge in the left nasal cavity. Oral examination showed absence of the second upper left premolar (**Fig. 9**), and a pus point along the left alveolar ridge. The ocular examination revealed no abnormality.

Incisional biopsy was taken from the alveolar region, and sent for KOH Staining, which revealed broad, aseptate hyphae, admixed with non-branching, thin filamentous structures.

**Fig. 10:**
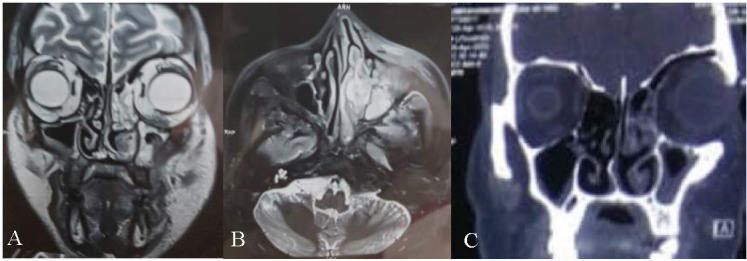
**A.** T2 weighted coronal section of MRI showing left frontal, ethmoidal and maxillary sinusitis with periantral extension into the premaxillary soft tissues, involving the left nasal cavity and the middle and inferior turbinates. **B.** axial MRI image showing involvement of the left infratemporal and pterygoid involvement along with ethmoidal and maxillary sinusitis. **C.** Coronal section of CT PNS showing moderate mucosal thickening in the inferior left frontal sinus, left maxillary and left ethmoidal sinus

CEMRI of PNS and orbits, showed left frontal, ethmoid and maxillary sinusitis, with peri-antral spread into left pre-maxillary soft tissues, left infratemporal and pterygoid fossa, medially extending into pterygopalatine foramen, and involving the left nasal cavity encasing the middle and inferior turbinates (**Fig. 10 A-C**). CT scan (PNS) showed bony erosions in the inferior wall of the left orbit, and moderate mucosal thickening of inferior left frontal sinus, left maxillary and ethmoid sinuses, and left ethmoidal recess.

**Fig. 11:**
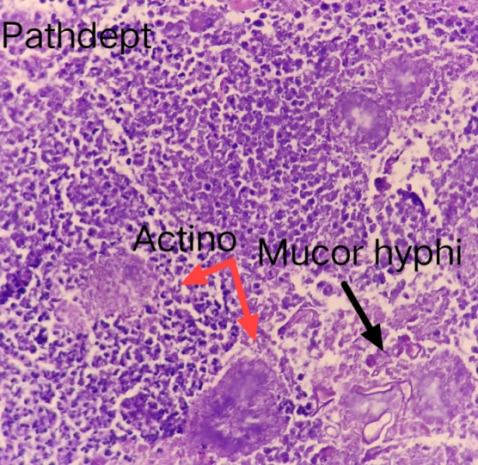
Actinomycotic colonies showing rosette shaped arrangement of filaments, along with aseptate, broad mucor hyphae, admixed with inflammatory granulation tissue

A diagnosis of invasive fungal sinusitis was made based on the above findings and injection Liposomal AMB (5 mg/ kg/ day) was started along with insulin infusion. A left extended partial maxillectomy was done and tissue was sent for histopathology, which showed numerous thick-walled branching aseptate hyphae of mucor admixed with inflammatory granulation tissue surrounding polymorphs and colonies with rosette-pattern arrangement of filaments, suggestive of Actinomycotic colonies along with Mucor (**Fig. 11**).

**Fig. 12:**
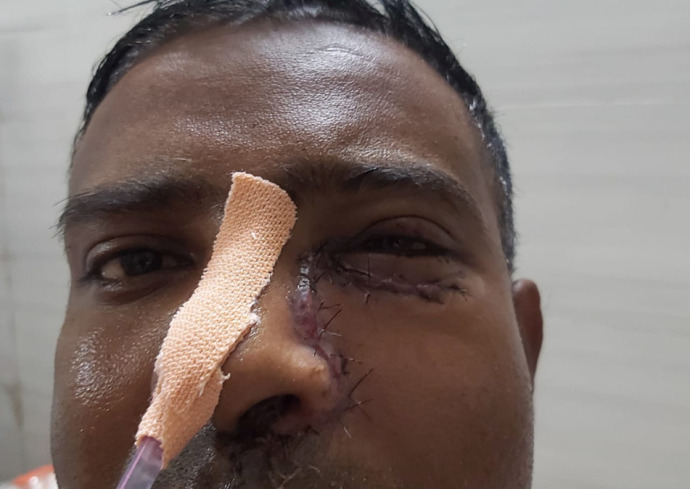
Second post-operative day

The patient did well post-operatively and was kept on intravenous (i.v.) antibiotics, and intravenous (i.v.) Liposomal AMB injections (**Fig. 12**). His serum electrolytes and RFT were monitored daily. He was discharged after receiving 30 doses of the injection.

## CASE 3

The patient, a 52-year-old male, presented to our hospital on 20th May, 2021, with the chief complaints of drooping of left upper eyelid, restriction of ocular movements and bulging of the left eye, which he noticed around 7 days before presentation, along with headache. His RTPCR showed that he was Covid positive. Upon admission, he had a normal lipid profile, KFT, serum LDH and serum procalcitonin levels along with normal serum electrolytes and normal IL-6 levels and RBS level. His CRP was reactive and raised 19.31 mg/ dl (<6). Australia antigen (HbsAg), anti-HCV were non-reactive, HbA1c was raised 8.9% (<6), and serum proteins were 5.31 g/ dl (<6.4). He received no dose of Covid vaccine.

**Fig. 13:**
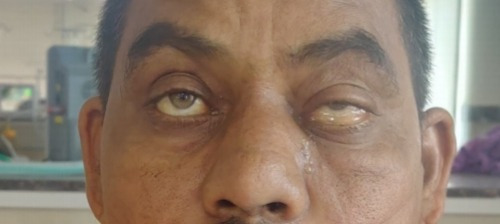
Severe ptosis and conjunctival chemosis

The visual acuity (VA) in both the right and left eyes was 20/ 80. Ocular movements were restricted in gazes in the LE and full in RE. Fundus examination showed a cup-disc ratio of 0.4 in the RE and 0.5 in the LE. Pupillary reactions were brisk in right and sluggish in LE, along with severe ptosis and conjunctival chemosis of LE (**Fig. 13**). Hertel’s exophthalmometry revealed 18 mm (RE) and 21 mm (LE).

**Fig. 14:**
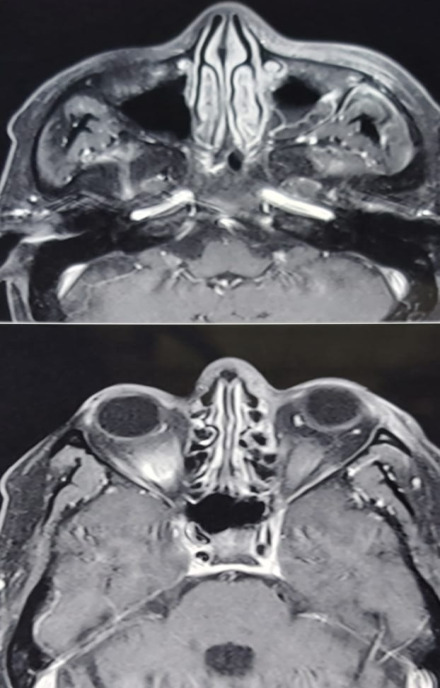
Axial section of MRI brain, PNS and orbits showing moderate mucosal thickening of the left maxillary sinus and both the ethmoidal sinuses. Thickened left optic nerve sheath can also be seen

**Fig. 15:**
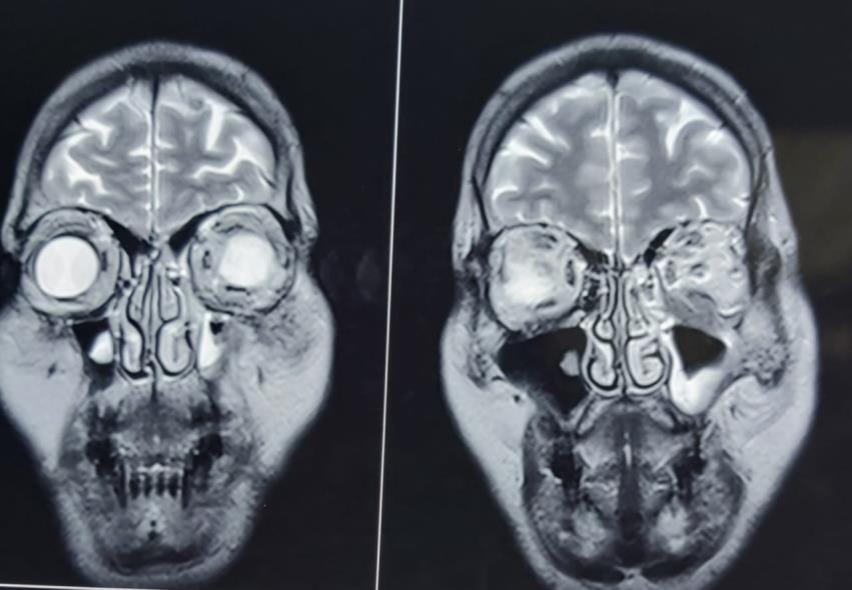
Coronal section of MRI PNS shows moderate mucosal thickening of the maxillary sinus along with soft tissue in left sided middle ethmoidal air cells, eroding the medial orbital wall. Hypertrophied middle and inferior turbinates can also be seen

There was bilateral hypertrophy of inferior turbinates and crusting in the nasal cavity. He was put on broad spectrum intravenous (i.v.) antibiotics. The CEMRI (PNS, Orbits, and brain) showed moderate mucosal thickening of the left maxillary sinus and of both ethmoid sinuses along with soft tissue in left sided middle ethmoid air cells, eroding the medial left orbital wall. The lesion was seen extending to peri-antral region and involving the left orbital apex and optic nerve sheath. The findings were suggestive of rhino-orbital mucormycosis (**Fig. 14**,**15**). His RT-PCR test was negative on 26.05.21.

**Fig. 16:**
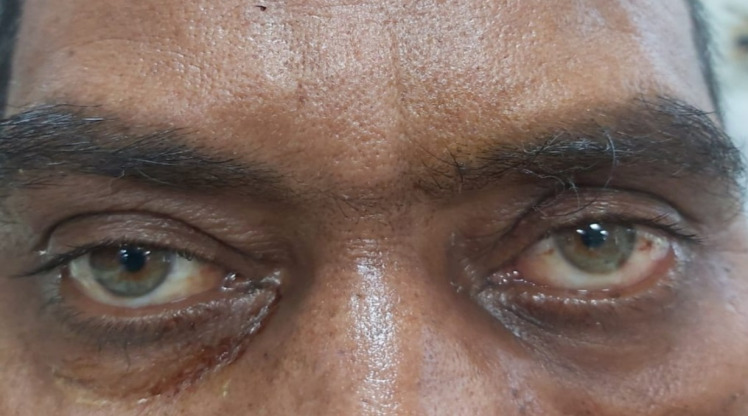
Significant improvement in ptosis on day 3 after FESS and TRAMB

3.5 mg of transcutaneous retrobulbar injection of liposomal Amphotericin B (TRAMB) was administered in LE and a functional endoscopic sinus surgery (FESS) was performed in the same sitting. Two days post-injection, his VA in the LE improved to 20/ 30 and a significant improvement in his ptosis (**Fig. 16**) was observed. The ocular motility in the LE remained restricted in all the gazes. TRAMB was repeated on days 8, 10, 12 and 14 after the first injection, but there was no further improvement in the VA and the ocular movements, which remained restricted. 

**Fig. 17:**
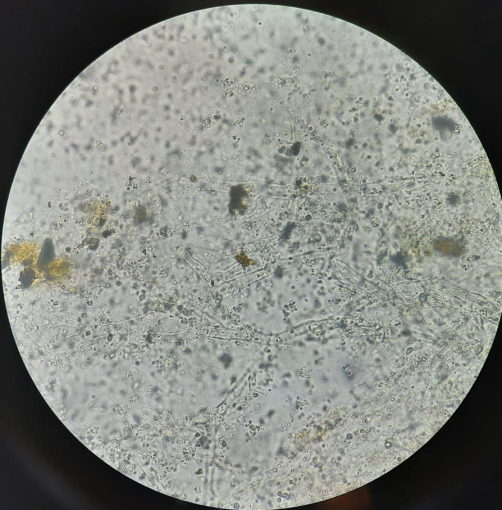
KOH mount (40X) showing broad, aseptate hyphae of mucor

Post-operatively, he was kept on liposomal AMB along with intravenous (i.v.) antibiotics. His KOH mount (**Fig. 17**) showed broad, aseptate hyphae of mucor. He showed good post-operative recovery with maintained serum electrolytes and RFT. He was discharged after receiving 30 doses of liposomal AMB injection.

## CASE 4

The patient, a 32-year-old male, presented to our facility on 14th May 2021 with the chief complaints of nasal stuffiness of left nostril, and difficulty in breathing during sleep, along with headache for 3 days. 23 days prior to this, he was diagnosed Covid positive on RT-PCR. He was admitted in a district level Covid hospital and his admission records of the time showed that he had high blood sugar levels (>380 mg/ dl), which were finally controlled on insulin. Before, he had also received injection dexamethasone (4 mg) BD for 5 days. After 5 days, he was put on oral methylprednisolone (32 mg) BD for 1 day, then 16 mg BD for 4 days, and subsequently 8 mg BD for 2 days. He was on supplemental oxygen therapy for 8 days. He was not vaccinated for Covid. His admission RBS was 178.27 mg/ dl (<140), admission blood urea nitrogen (BUN) was 28.56 mg/ dl (6-21) and serum urea was 61.11 mg/ dl (13-43). Serum lactate dehydrogenase (LDH) was 761.1 U/ l (240-480). The LFT showed increased serum alkaline phosphatase (ALP) 271.14 U/ l (<270), serum aspartate transaminase (AST) 263.48 U/ l (<35), serum alanine transaminase (ALT) 551.84 U/ l (0-45). The coagulation profile and CRP were within normal limits. Australia antigen HbsAg, anti-HCV, HIV1 and HIV2 were non-reactive. Total leucocyte count (TLC) was 12800 cells/ mm3 (4000-11000). The HbA1c was 9.8%.

**Fig. 18:**
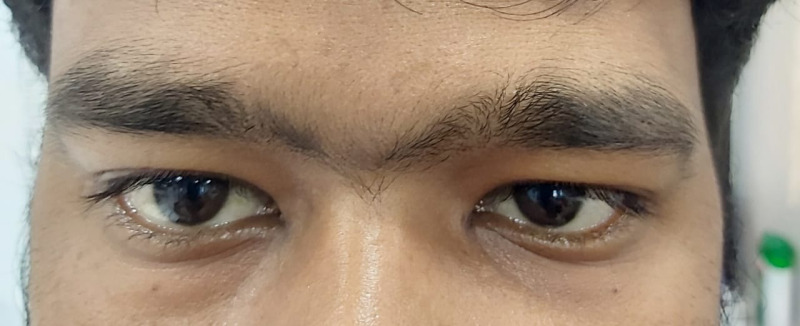
No ocular abnormality

**Fig. 19:**
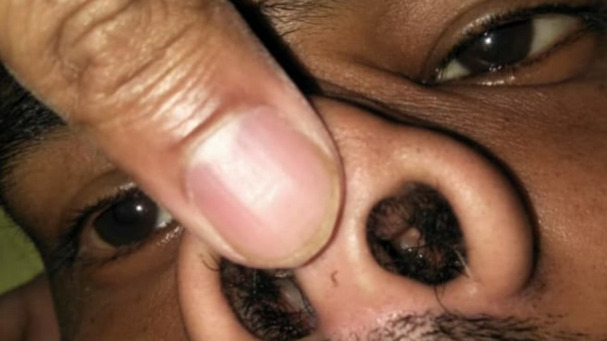
Black debris in the left nostril

The ocular adnexa and the anterior segment were within normal limits (**Fig. 18**). The VA was 20/ 30 in both eyes. Fundus examination of the RE showed a few soft exudates 1/ 3rd disc diameter below the inferotemporal arcade. The nasal cavity showed blackish debris in the left nostril (**Fig. 19**).

**Fig. 20:**
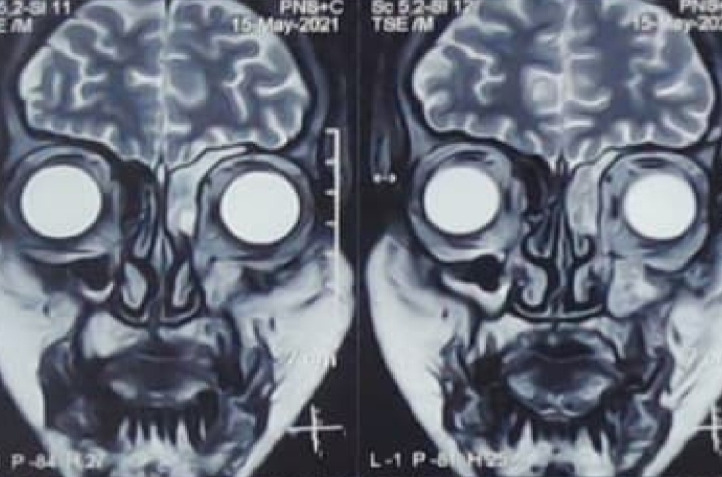
Coronal section of CEMRI PNS showed mucosal thickening of both maxillary (left more than right), ethmoid sinuses with retained secretions

**Fig. 21:**
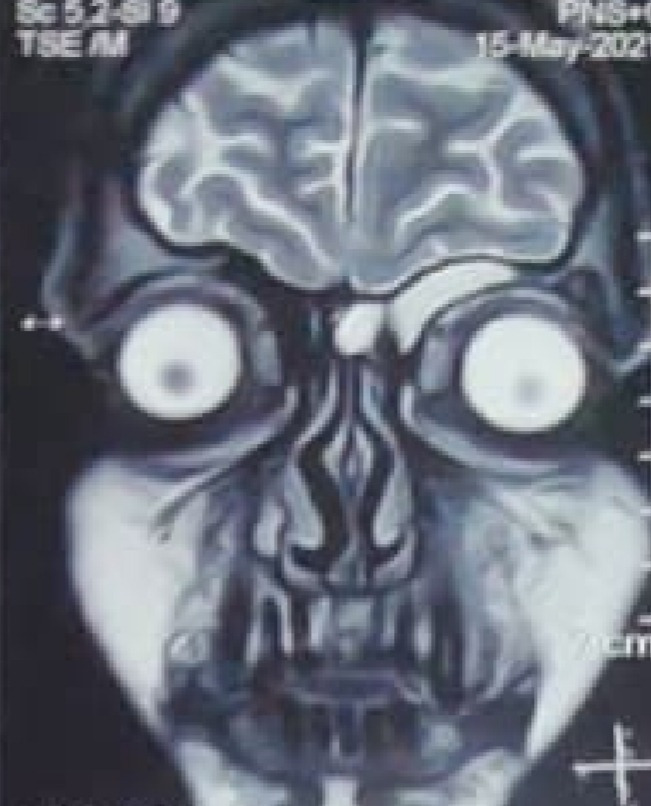
Coronal section of CEMRI PNS showing frontal sinuses with retained secretions

CEMRI of the PNS showed mucosal thickening in both maxillary (left more than right), ethmoid sinuses with retained secretions with bulging of the medial wall/ lamina papyracea of the orbit (**Fig. 20**,**21**). Corroborative CT of PNS showed erosion of the medial wall of left maxillary sinus. No obvious extension in the orbit or the cranium was seen. 

**Fig. 22:**
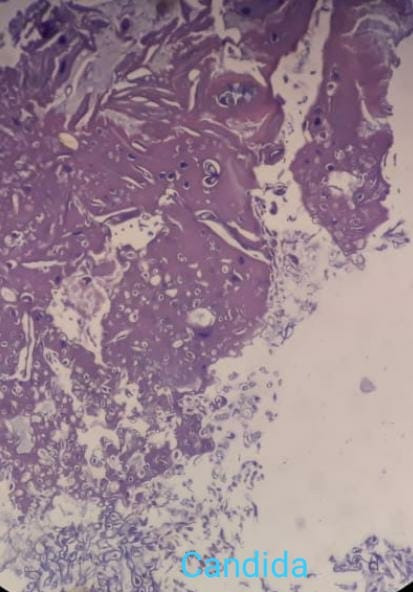
H and E-stained slide (40X) showing broad, aseptate mucor hyphae along with Candida spores

Based on the above reports, the patient was taken in for an early functional endoscopic sinus surgery (FESS) with debridement and the tissue obtained was sent for histopathology. The hematoxylin and eosin (H&E) stained slide showed aseptate, broad hyphae branching at right angles, along with Candida spores (**Fig. 22**). 

The patient was started post-operatively on injection liposomal AMB along with 8 units of regular insulin injection. Injection Meropenem (500 mg) and injection Clindamycin (600 mg) were given TDS intravenously. After the histopathology confirmed Candida, injection Caspofungin was started intravenous (i.v.), 70 mg on day 1 and then 50 mg/ day for 14 days. The patient was discharged on the 14th post-operative day on tablet Posaconazole (300 mg/ day) and is currently being followed on an outpatient basis.

## CASE 5

The patient, a 45-year-old male, a known diabetic for 5 months, well controlled on oral hypoglycaemic drugs, presented to our post-Covid facility with chief complaints of headache, right sided facial pain, bilateral periorbital pain, and reduced vision in the right eye for 3 days. The patient had a history of Covid infection. He was tested positive on RT-PCR on 24th April 2021, after which he was admitted in a Covid treatment facility for further management. He received supplemental oxygen therapy and intravenous injection dexamethasone along with budesonide nebulization thrice a day during his treatment. His RT-PCR was negative on 11th May 2021, after which he was discharged. He was advised to take tablet Medrol 8 mg for a week, which he discontinued 5 days following discharge. Thereafter, he was readmitted to our facility with the above complaints on 22nd May, 2021. He received no dose of Covid vaccine.

Upon admission, his blood investigations revealed an RBS of 139 mg/ dl, HbA1c 13.1%, elevated VLDL cholesterol 41 mg/ dl (12-32 mg/ dl). HIV1, HIV2, Australia Antigen and Anti-HCV antibodies were non-reactive, CRP was reactive, serum LDH was 598.4 U/ l (240-480 U/ l), serum K+ 3.19 mmol/ l (3.5-5.3 mmol/ l) indicating hypokalemia. LFT, RFT and coagulation profile were normal, and lymphocytes were 11.3% (moderate lymphopenia) along with low hematocrit of 35.5% (38-45%).

**Fig. 23:**
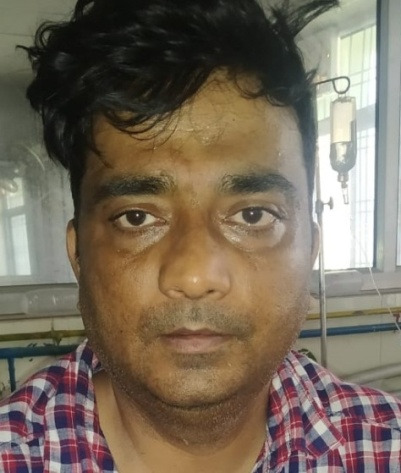
Right sided facial swelling

Upon physical examination, a mild facial swelling on the right side of the face and tenderness over the right cheek and the right periorbital region (**Fig. 23**) were observed. The ocular and nasal cavity examinations were unremarkable.

**Fig. 24:**
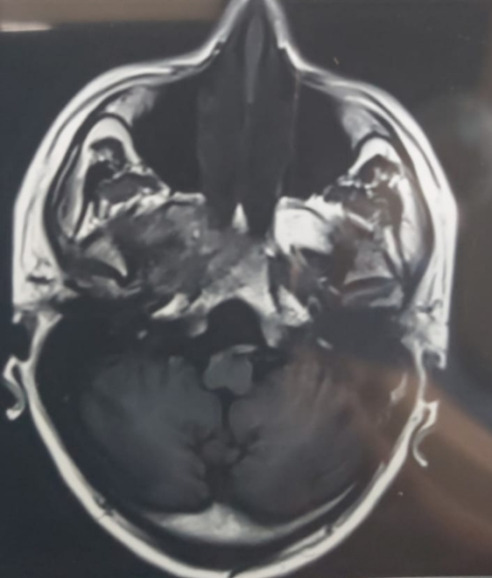
Axial MRI image showing extension into infratemporal fossa and periantral extension

CEMRI of PNS and orbits revealed bilateral ethmoid, sphenoid, and right frontal sinusitis with periantral extension and involvement of right infratemporal fossa, along with bilateral inferior and middle turbinates hypertrophy (**Fig. 24**).

**Fig. 25:**
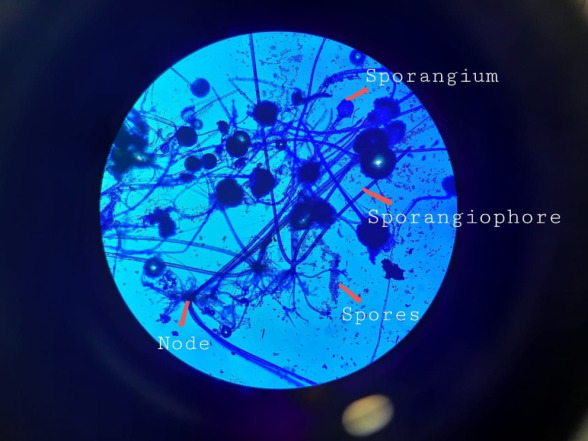
Lactophenol cotton blue staining (10X) showing Rhizopus characterized by presence of sporangiophores arising from nodes directly above the rhizoids and multispored sporangia

Functional Endoscopic Sinus Surgery (FESS) was performed to debride the tissues and to obtain samples for histopathological examination. Lactophenol cotton blue staining showed the presence of Rhizopus species characterized by the presence of sporangiophores arising from nodes directly above the rhizoids and globose sporangium that were multispored (**Fig. 25**).

A diagnosis of invasive fungal sinusitis was made and injection liposomal AMB (5 mg/ kg/ day) was started along with insulin infusion.

**Fig. 26:**
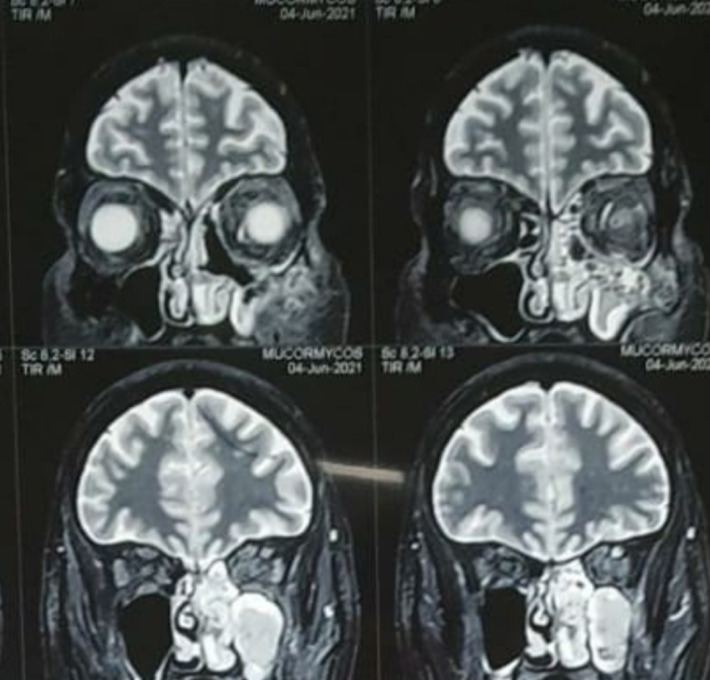
T2 weighted coronal section image showing soft tissue in the left maxillary sinus and fluid intensity signal in the left maxillary sinus

The patient did well post FESS and was kept on i.v. antibiotics and i.v. liposomal Amphotericin B (AMB) along with tab. Posaconazole 300 mg once a day. His serum electrolytes and RFT were monitored daily. TRAMB was performed in the right eye. An MRI scan was done following the FESS and TRAMB revealed soft tissue, along with fluid intensity signal in the left maxillary sinus (**Fig. 26**).

A repeated endoscopy was performed on the patient 14 days later and the sample taken for histopathology revealed no fungal elements, thus concluding that the disease had been treated successfully and the patient was discharged on oral Posaconazole (300 mg).

## CASE 6

A 22-year-old male presented to the triage facility of our hospital on 24.05.21, with complaints of headache and fever for 9 days, and drooping of the right eyelid and loss of vision in the RE for 5 days.

His RT-PCR was positive for Covid and he was shifted to Covid ward. Along with i.v. antibiotics, he also received supplemental oxygen for 5 days and tab Medrol 4 mg for 4 days. He was non-vaccinated for Covid-19.

His admission RBS was 290 mg% and the complete blood counts showed decreased polymorphs 35 (50-70%) and increased lymphocytes. The D-Dimer was 540 ng/ ml (<500 ng/ ml). His LFT and RFT and lipid profile were within normal limits. The HbsAg, anti-HCV, HIV1 and HIV2 were non-reactive. The CRP was raised - 40.51 mg/ dl (<6).

**Fig. 27:**
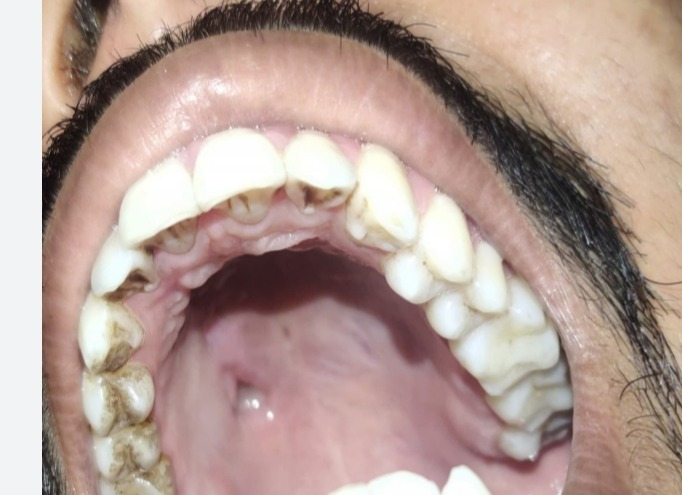
Ulcer (2×2 cm) on hard palate

Oral cavity examination showed an isolated ulcer (2 cm × 2 cm) on the hard palate (**Fig. 27**).

**Fig. 28:**
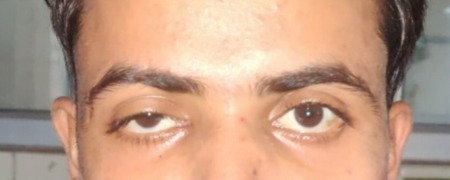
Mild ptosis in right eye

Mild ptosis (**Fig. 28**) and mid-dilated pupil with a relative afferent pupillary defect was present in the RE. The optic disc of the RE showed a cup-disc ratio of 0.5 in RE and 0.3 in LE. An inferior bullous retinal detachment was present and no perception of light in the RE was observed. The left ocular examination was within normal limits and the VA was 20/ 30 in the LE.

**Fig. 29:**
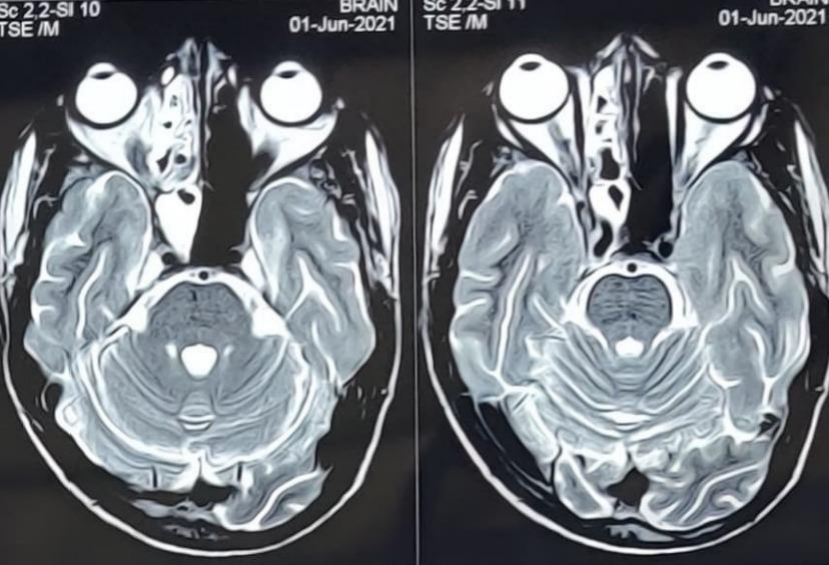
Axial section of MRI showing thickened optic nerve sheath in the right eye and involvement of the right ethmoidal air cells

**Fig. 30:**
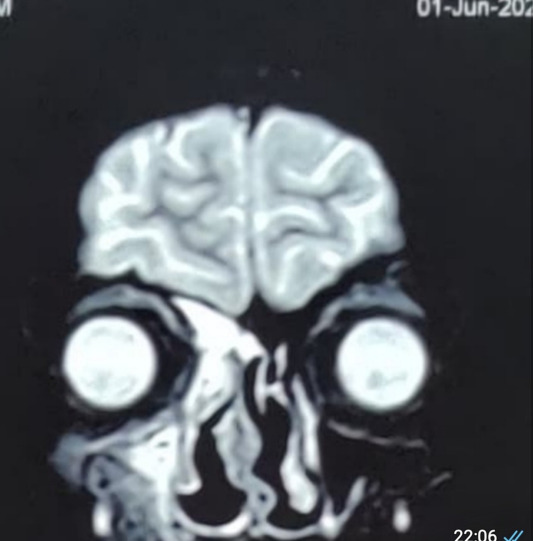
T2 weighted coronal section on MRI showing involvement of the right frontal and maxillary sinuses

**Fig. 31:**
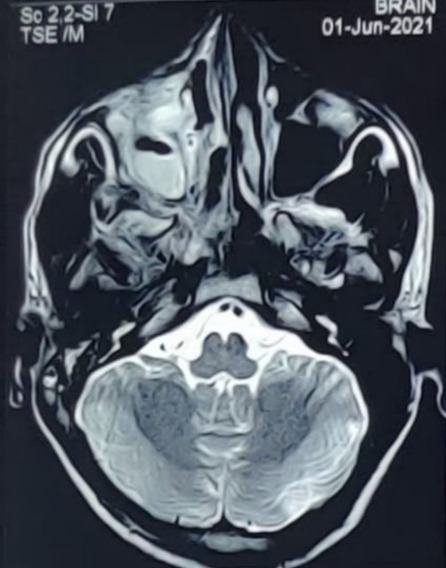
MRI axial section showing marked mucosal thickening of the right maxillary sinus

MRI of brain, PNS and orbits with Magnetic Resonance Angiography (MRA) showed right sided rhino-orbital mucormycosis involving right maxillary, ethmoid and sphenoid sinuses extending in anterior and posterior peri-antral region, right orbital apex with thickened optic nerve sheath and extension of lesion in ipsilateral pterygomaxillary, inferior orbital fissure, pterygopalatine and infratemporal fossa. MRA (brain) did not reveal any abnormality (**Fig. 29**-**31**).

**Fig. 32:**
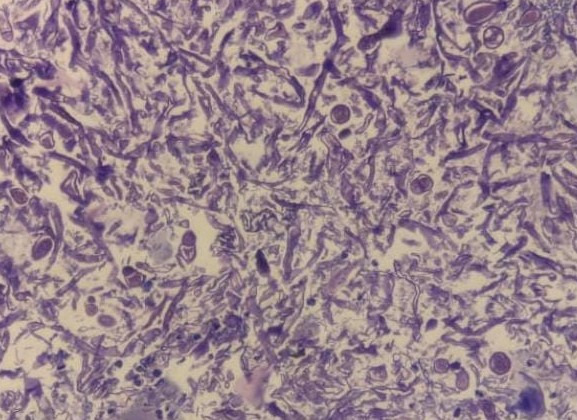
H & E-stained slide (x40) showing broad, aseptate, mucor hyphae along with Cryptococcus spores

**Fig. 33:**
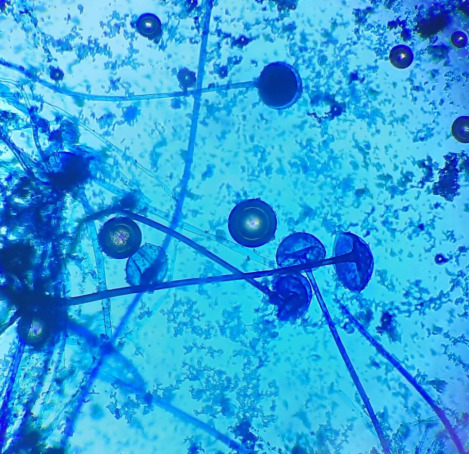
Lactophenol cotton blue staining (x40) showing mucor

Based upon the MRI findings, a presumptive diagnosis of rhino-orbital mucormycosis was made and tablet Medrol was withdrawn from the therapeutic regimen. TRAMB was done in the right eye and (i.v) liposomal AMB was started. His RT-PCR highlighted a Covid-negative result on 01.06.21, after which a FESS was done. Tissue retrieved was sent for histopathology and showed Cryptococcus spores along with broad aseptate mucor hyphae (**Fig. 32**). The lactophenol cotton blue stain also showed mucor (**Fig. 33**).

Post-operatively, he was started on tazobactam and piperacillin (4.5 gm 6 hourly) and AMB. After Cryptococcus was confirmed on histopathology, injection Caspofungin was added to the treatment regimen (70 mg i.v. on first day followed by 50 mg once daily). Serum creatinine increased to 2.77 mg/ dl after 6 days. A nephrology reference mandated stopping of i.v. liposomal AMB. This was substituted with i.v. Posaconazole (300 mg on the first day, followed by 100 mg on subsequent days). Doses of tazobactam and piperacillin were also reduced. His RFT finally came normal after 7 days. 

**Fig. 34:**
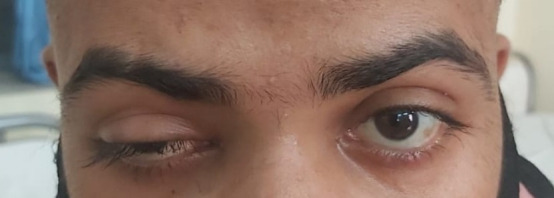
Before second injection of TRAMB

**Fig. 35:**
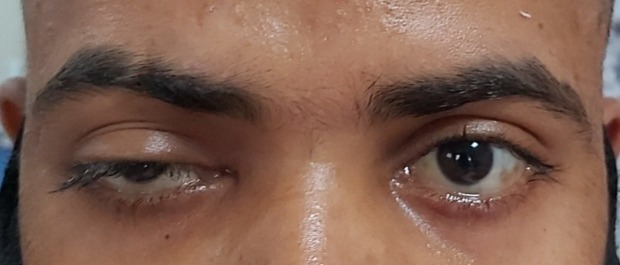
Marginal improvement in ptosis (OD) after second injection of TRAMB

A repeated endoscopy was done after 2 weeks of surgery and the KOH staining of the tissue did not show any fungal elements. TRAMB was repeated on 11.06.2021. On the 2nd post-injection day, his ptosis seemed to have improved marginally (**Fig. 34**,**35**). TRAMB was repeated on days 10, 12 and 14. No further improvement in the ptosis was noted. He was kept on i.v. liposomal AMB and tablet Posaconazole (300 mg OD) and his serum electrolytes and RFT were monitored daily. He was discharged after having received 30 injections of i.v. liposomal AMB.

## Discussion

Mucormycosis are a range of infections that are caused by fungi known as Zygomycetes. These saprophytic fungi are freely found in soil, bread, air, dust, hospital ward rooms [**13**,**14**]. 

A recent study in India [**15**] on mucormycosis cases showed the use of corticosteroids (either oral or intravenous or both) as the most common risk factor. Immunocompromised status of the host due to factors like diabetes mellitus, immunosuppressive therapy, leukemias, neutropenias and malnourishment also serve as important risk factors for the disease [**16**]. Diabetic ketoacidosis, hematopoietic stem cell transplantation, HIV/ AIDS and iron overload are some other important risk factors. 

According to an Indian study, Rhizopus oryzae is the most common isolate followed by Apophysomyces elegans [**17**]. Other fungi implicated are Mucor spp., Saksenaea, and Cunninghamella [**18**]. 

According to the anatomical site of involvement, mucormycosis can be rhino cerebral, pulmonary, cutaneous, gastrointestinal, and disseminated. Rhino-orbital-cerebral mucormycosis (RCOM) is the most common presentation in mucormycosis, comprising about two-thirds of all its cases [**19**]. An immunocompetent person who inhales these fungal spores or if they gain entry through the oral cavity, the phagocytic response in the body limits their spread and any harm caused by the fungus. But, in an immunocompromised host, these spores are deposited on the mucosa of the nasal turbinates, where they germinate, develop hyphae, and subsequently infect the paranasal sinuses, the orbits and the brain. The fungus reaches the cranium either through the orbital apex or breach in cribriform plate of the ethmoid bone, or through spreading within the vasculature. This is an aggressive angioinvasive phenomenon. The fungus proliferates within the internal elastic lamina of the vessel, giving rise to thromboembolisms and infarction and necrosis of the involved tissues.

DKA is the most common risk factor. Ketone bodies found in this condition allow the fungi to produce ketoreductase, which helps in the growth of the fungus. Diabetes mellitus is associated with 40% of all mucormycosis cases and about 70% of cases of RCOM [**20**]. In our study, all patients were diabetic. 

The confirmation of the diagnosis is based on the histological analysis, culture, or wet mount characteristics of the tissue biopsy. Microscopic examination reveals broad, aseptate hyphae, which branch irregularly at right angles [**21**]. One patient’s histology revealed superadded actinomyces infection. One patient’s specimen showed Candida spores along with mucor hyphae and yet another’s showed the presence of Cryptococcus spores.

Most clinicians would start the treatment if the clinical picture of the patient is corroborated by a positive direct smear report because waiting for culture positivity may result in delay in initiating a timely treatment, which greatly increases the risk of mortality. Even the diagnostic nasal endoscopy can be used in RCOM suspects and is good for diagnostic sampling from the nasal cavity as well. It is helpful even before the clinical and radiological signs become evident. We performed a pre-operative endoscopy in two cases to directly receive the sample from the area of necrosis and in one of our patients post-operatively, to retrieve sample for histopathology again.

Radiological imaging plays a very important role in establishing the diagnosis and extent of the disease. A timely MRI of paranasal sinuses, the orbits, and the cranium can bring essential light on the extent and spread of this very aggressive infection. Although CEMRI was our preferred imaging modality, a contrast-enhanced CT scan is relatively faster and can be used in patients in whom MRI is not possible.

Clinical signs that are most common, include palatal ulceration or necrosis, a blood-stained nasal discharge, facial swelling, and black eschar in the nasal cavities [**13**]. Ocular signs most commonly reported were ophthalmoplegia, loss of vision, conjunctival chemosis and proptosis [**22**]. In our study, 33% of the cases presented with no ocular signs, and 100% of the cases had a history of headache. Also, one patient presented with the history of loss of 1 tooth. 

Almost all patients in our study presented within 12 days of the onset of their symptoms, and two-thirds presented within the first 6 days. Prompt institution of treatment in the form of liposomal AMB and surgery was lifesaving in them. In our series, a timely treatment was initiated in all patients and surgery was offered. Literature shows that patients whose treatment is started within 6 days have a survival rate of 76-81% and if delayed to more than 12 days, this rate drops down to 36-42%. One of our patients, who requested an early discharge 25 days post maxillectomy surgery, died.

AMB dramatically increases the survival rate to 60% [**23**] and constitutes the first line medical therapy for the disease. This is continued for at least six weeks in the dosage of 5-10 mg/ kg body weight per day. During the therapy, stringent monitoring of the RFT and serum electrolytes is recommended. However, its liposomal preparation is considered much safer.

If a person develops nephrotoxicity on AMB, Posaconazole can be reconstituted. In our case series, only one patient needed to be administered i.v. Posaconazole. Also, 2 patients developed hypokalaemia, and were offered corrective therapy. In these two, liposomal AMB was replaced by i.v. Posaconazole only for a short duration and after a medicine reference, liposomal AMB was restarted along with potassium supplementation, when their serum electrolytes showed a normal serum potassium level. All patients were discharged on oral Posaconazole (300 mg) once a day. 

The blood sugar levels were monitored thrice daily in all patients and all were kept on insulin. It is a remarkable fact that all our study subjects had either a history of DM or developed increased sugar levels while on treatment with oral steroids during their Covid phase. Published literature [**19**] shows that the history of systemic steroid use was found in 76% of patients with Covid-19 associated RCOM. In India, this fraction is still higher [**24**]. So, injudicious use of corticosteroids can be a possible cause for RCOM. DM itself has been identified as an independent risk factor in mucormycosis [**25**]. 

Surgical debridement of necrotic tissue, sometimes repeatedly, may be necessary to ensure no further progression of the disease. All our patients underwent a surgical procedure (2 underwent maxillectomy and 4 had FESS). 

TRAMB has been reported as a minimally invasive and globe sparing intervention for orbital disease. We gave injections in two of our patients who had a confirmed orbital involvement and used the liposomal preparation of AMB for that. This injection may be a viable therapeutic option in those cases in which either the burden of orbital disease is not substantial or the patient refuses consent for exenteration due to cosmetic disfigurement that ensues. One patient refused exenteration and his perception of light was negative, so he was managed with repeated injections of TRAMB [**26**] (on days 1, 8, 10, 12, 14). The intraocular pressures in both patients were slightly raised (21-25 mmHg), which were normalized in one day with orally given carbonic anhydrase inhibitor and were continued for 3 days post TRAMB. One of the patients showed a retro-orbital extension despite repeated TRAMB injections. What should be mentioned is that although orbital exenteration could be lifesaving, the decision to exenterate is the most difficult one for the treating ophthalmologist. 

All patients were followed on an outpatient basis and mortality occurred only in one case. Early recognition and timely initiation of medical and surgical therapy along with a good glycemic control form the basic crux of the treatment of mucormycosis. 

## Study Limitations

Our data is limited and is representative of the cases admitted in our Institute. Most of them are in active follow-up. Therefore, the data needs to be interpreted with caution and we hope it may provide some insight into the management of the patients.

## Conclusion

There has been a surge in the number of mucormycosis cases in the Covid era. This can be attributed to supplemental oxygen therapy, indiscriminate use of steroids, or prolonged admission in hospital wards. Although the exact etiology remains unclear, a state of prevailing hyperglycemia seems to serve as an important association. A high index of suspicion must be maintained for all such cases and early intervention plays a very important part. Timely institution of anti-fungal treatment along with a debridement surgery and controlling the blood sugar are the mainstay of management. Also, patients who have been put on immunosuppressives need to be monitored vigilantly. 

**Conflict of Interest statement**

Authors state no conflict of interest.

**Informed Consent and Human and Animal Rights statement**

Informed consent has been obtained from all individuals included in this study.

**Authorization for the use of human subjects**

Ethical approval: The research related to human use complies with all the relevant national regulations, institutional policies, is in accordance with the tenets of the Helsinki Declaration, and has been approved by the review board of Moti Lal Nehru Medical College, Prayagraj, Uttar Pradesh, India.

**Acknowledgements**

None.

**Sources of Funding**

None.

**Disclosures**

None.

## References

[1] Viterbo S, Fasolis M, Garzino-Demo P, Griffa A, Boffano P, Iaquinta C, Tanteri G, Modica R (2011). Management and outcomes of three cases of rhinocerebral mucormycosis. Oral Surg Oral Med Oral Pathol Oral Radiol Endod.

[2] Torres-Narbona M, Guinea J, Muñoz P, Bouza E (2007). Zygomycetes and zygomycosis in the new era of antifungal therapies. Rev Esp Quimioter.

[3] Afroze SN, Korlepara R, Rao GV, Madala J (2017). Mucormycosis in a Diabetic Patient: A Case Report with an Insight into Its Pathophysiology. Contemp Clin Dent.

[4] Neville WB, Damm D, Allen CM, Bouquet JE (2001). Textbook of Oral and Maxillofacial Pathology.

[5] Horby P, Lim WS, Emberson JR, Mafham M, Bell JL, Linsell L, Staplin N, Brightling C, Ustianowski A, Elmahi E, Prudon B, Green C, Felton T, Chadwick D, Rege K, Fegan C, Chappell LC, Faust SN, Jaki T, Jeffery K, Montgomery A, Rowan K, Juszczak E, Baillie JK, Haynes R, Landray MJ (2021). Dexamethasone in Hospitalized Patients with Covid-19. N Engl J Med.

[6] Jose A, Singh S, Roychoudhury A, Kholakiya Y, Arya S, Roychoudhury S (2021). Current Understanding in the Pathophysiology of SARS-CoV-2-Associated Rhino-Orbito-Cerebral Mucormycosis: A Comprehensive Review. J Maxillofac Oral Surg.

[7] Jeong W, Keighley C, Wolfe R, Lee WL, Slavin MA, Kong DCM, Chen SC (2019). The epidemiology and clinical manifestations of mucormycosis: a systematic review and meta-analysis of case reports. Clin Microbiol Infect.

[8] Chander J, Kaur M, Singla N, Punia RPS, Singhal SK, Attri AK, Alastruey-Izquierdo A, Stchigel AM, Cano-Lira JF, Guarro J (2018). Mucormycosis: Battle with the Deadly Enemy over a Five-Year Period in India. J Fungi (Basel).

[9] Chakrabarti A, Singh R (2014). Mucormycosis in India: unique features. Mycoses.

[10] Safar A, Marsan J, Marglani O, Al-Sebeih K, Al-Harbi J, Valvoda M (2005). Early identification of rhinocerebral mucormycosis. J Otolaryngol.

[11] Werthman-Ehrenreich A (2021). Mucormycosis with orbital compartment syndrome in a patient with COVID-19. Am J Emerg Med.

[12] Deutsch PG, Whittaker J, Prasad S (2019). Invasive and Non-Invasive Fungal Rhinosinusitis-A Review and Update of the Evidence. Medicina (Kaunas).

[13] Kolekar JS (2015). Rhinocerebral mucormycosis: a retrospective study. Indian J Otolaryngol Head Neck Surg.

[14] Safi M, Ang MJ, Patel P, Silkiss RZ (2020). Rhino-orbital-cerebral mucormycosis (ROCM) and associated cerebritis treated with adjuvant retrobulbar amphotericin B. Am J Ophthalmol Case Rep.

[15] Patel A, Kaur H, Xess I, Michael JS, Savio J, Rudramurthy S, Singh R, Shastri P, Umabala P, Sardana R, Kindo A, Capoor MR, Mohan S, Muthu V, Agarwal R, Chakrabarti A (2020). A multicentre observational study on the epidemiology, risk factors, management and outcomes of mucormycosis in India. Clin Microbiol Infect.

[16] Talmi YP, Goldschmied-Reouven A, Bakon M, Barshack I, Wolf M, Horowitz Z, Berkowicz M, Keller N, Kronenberg J (2002). Rhino-orbital and rhino-orbito-cerebral mucormycosis. Otolaryngol Head Neck Surg.

[17] Chakrabarti A, Chatterjee SS, Das A, Panda N, Shivaprakash MR, Kaur A, Varma SC, Singhi S, Bhansali A, Sakhuja V (2009). Invasive zygomycosis in India: experience in a tertiary care hospital. Postgrad Med J.

[18] Chakrabarti A (2010). Cutaneous zygomycosis: major concerns. Indian J Med Res.

[19] Singh AK, Singh R, Joshi SR, Misra A (2021). Mucormycosis in COVID-19: A systematic review of cases reported worldwide and in India. Diabetes Metab Syndr.

[20] Ribes JA, Vanover-Sams CL, Baker DJ (2000). Zygomycetes in human disease. Clin Microbiol Rev.

[21] Walsh TJ, Gamaletsou MN, McGinnis MR, Hayden RT, Kontoyiannis DP (2012). Early clinical and laboratory diagnosis of invasive pulmonary, extrapulmonary, and disseminated mucormycosis (zygomycosis). Clin Infect Dis.

[22] Bhansali A, Bhadada S, Sharma A, Suresh V, Gupta A, Singh P, Chakarbarti A, Dash RJ (2004). Presentation and outcome of rhino-orbital-cerebral mucormycosis in patients with diabetes. Postgrad Med J.

[23] Petrikkos GL (2009). Lipid formulations of amphotericin B as first-line treatment of zygomycosis. Clin Microbiol Infect.

[24] John TM, Jacob CN, Kontoyiannis DP (2021). When Uncontrolled Diabetes Mellitus and Severe COVID-19 Converge: The Perfect Storm for Mucormycosis. J Fungi (Basel).

[25] Jeong W, Keighley C, Wolfe R, Lee WL, Slavin MA, Kong DCM, Chen SC (2019). The epidemiology and clinical manifestations of mucormycosis: a systematic review and meta-analysis of case reports. Clin Microbiol Infect.

[26] Hirabayashi KE, Kalin-Hajdu E, Brodie FL, Kersten RC, Russell MS, Vagefi MR (2017). Retrobulbar Injection of Amphotericin B for Orbital Mucormycosis. Ophthalmic Plast Reconstr Surg.

